# Response of a Pioneering Species (*Leptospermum scoparium* J.R.Forst. & G.Forst.) to Heterogeneity in a Low-Fertility Soil

**DOI:** 10.3389/fpls.2019.00093

**Published:** 2019-02-06

**Authors:** Maria Jesus Gutiérrez-Ginés, Engracia Madejón, Niklas J. Lehto, Roger D. McLenaghen, Jacqui Horswell, Nicholas Dickinson, Brett H. Robinson

**Affiliations:** ^1^Faculty of Agriculture and Life Sciences, Lincoln University, Lincoln, New Zealand; ^2^The Institute of Environmental Science and Research Ltd., Christchurch, New Zealand; ^3^The Institute of Natural Resources and Agrobiology of Seville, Spanish National Research Council (CSIC), Seville, Spain; ^4^College of Health, Massey University, Wellington, New Zealand; ^5^School of Physical and Chemical Sciences, University of Canterbury, Christchurch, New Zealand

**Keywords:** biosolids, mānuka, nutrient patches, plant nutrition, rhizobox, root foraging, soil heterogeneity

## Abstract

Root foraging may increase plant nutrient acquisition at the cost of reducing the total volume of soil explored, thereby reducing the chance of the roots encountering additional patches. Patches in soil seldom contain just one nutrient: the patch may also have distinct textural, hydrological, and toxicological characteristics. We sought to determine the characteristics of root foraging by a pioneering species, *Leptospermum scoparium*, using pot trials and rhizobox experiments with patches of biosolids. The growth of *L. scoparium* was increased by <50 t/ha equiv. of biosolids but higher doses were inhibitory. Roots foraged patches of biosolids in a low-fertility soil. There was no evidence of chemotaxis, rather, the roots proliferated toward the patch of biosolids, following chemical gradients of nitrate. While the biosolids also contained high concentrations of other nutrients (P, K, and S), only significant chemical gradients of nitrate were found. Once the roots encountered a patch of biosolids, the growth of the plant increased to a level similar to plants growing in soil homogeneously mixed with biosolids or surface-applied biosolids. Our results indicate that roots forage nitrate, which is mobile in soil, and that gradients of nitrate may lead to patches containing other less mobile nutrients, such as phosphate or potassium.

## Introduction

Plants respond to nutrient deficiencies in soil by increasing the root: shoot biomass ratio (Marschner, 2012). However, there are contrasting responses of roots when the distribution of nutrients in the soil is heterogeneous. Some plants respond to this heterogeneity by proliferating roots into or foraging roots toward a nutrient-rich zone, or by increasing their physiological capacity for taking up those nutrients (Hodge, 2004, 2009). Hodge (2009) reviewed studies investigating the characteristics, and triggers of root behaviors. Given limited resources of assimilate, plants may allocate resources to a root system that explores a large volume of soil or use those resources to proliferate roots in patches that are rich in nutrients. Foraging behavior may be morphological or physiological. There is no consistency between species in their responses to soil heterogeneity. Within a species, root responses to heterogeneity depend on environmental factors, including soil type, the geometric distribution of nutrient-rich patches, as well as the type of nutrients present in the patch (Hodge, 2004, 2009). McNickle et al. (2009) suggested the use of an optimality framework to explain the root foraging behavior of plants, which could help to explain, and unify the contrasting results that have been obtained in this research field.

High concentrations of some trace elements or other toxic agents in soils can also induce variations in root architecture (Arduini et al., 1994). Roots can respond to these toxic agents by either tolerance, avoidance, or inhibition. Plants can tolerate toxic agents in the soil by reducing their bioavailability through root exudates or recruiting specialist microorganisms to that end (Dimkpa et al., 2009), preventing their entry into symplast, and actively transporting toxins from the symplast into the apoplast (Robinson et al., 2009). Root architecture may change, resulting in fewer roots growing near or in a patch containing toxic agents (Moradi et al., 2009; Robinson et al., 2009; Khare et al., 2017). As with nutrients, plant responses to toxic agents depend on their distribution in soil (Solomon-Wisdom et al., 2015).

Most of the experiments for studying the foraging behavior of root systems are carried out in plates with artificial growth media (Khare et al., 2017) or with nutrient solution either in pots (Hodge, 2009), or in rhizoboxes. Reis et al. (2017) used rhizoboxes (15 cm × 30 cm × 2 cm) filled with sand; Moradi et al. (2009) had rhizoboxes that were 17 cm × 15 cm × 1.5 cm, filled with sand and irrigated with nutrient solution. The above-mentioned studies all propounded the necessity of further research on root behavior in contrasting scenarios, for example with a stratified soil profile and a growth period of months rather than weeks.

*Leptospermum scoparium* is a member of the Myrtaceae family and one of the most widely distributed, abundant, and hardy member of the New Zealand indigenous woody flora (Stephens et al., 2005). It is a pioneer species that often colonizes environments where climax forest cannot develop due to extreme wet, dry, cold, exposed, infertile, and/or unstable soils (Stephens et al., 2005). The root system of *L. scoparium* consists on a tap root with a few main structural roots of small diameter that give rise to a dense network of fine roots. The lateral roots are mainly concentrated in the top 20 cm of soil and the roots that extended furthest are generally within 10 cm of the soil surface (Watson and O’Loughlin, 1985). These authors reported that >96% of the total root length of *L. scoparium* consisted of roots with diameter <20 mm and these fine roots comprised just 30% of the root biomass.

Reis et al. (2017) reported that when grown in sand, *L. scoparium* roots foraged patches of biosolids (dewatered pond sludge). Biosolids addition to soils can increases the accumulation of trace elements in leaves and stems of *L. scoparium* (Reis et al., 2017). However, even a large biosolids application (1350 kg N ha^−1^ equiv.) to rebuild a low-fertility soil did not result in phytotoxic concentrations in this species (Esperschuetz et al., 2017a,b).

There is a significant knowledge gap on whether plants in general and *L. scoparium* in particular, have a mechanism where a root entering a patch of nutrients signals to other roots to grow toward that patch (Ruffel et al., 2011) or whether roots simply proliferate within a patch or along a chemical gradient in soil.

Given that it is not possible to create a nutrient patch in soil without changing other soil parameters, we used biosolids (treated sewage sludge) to represent what may occur both naturally (i.e., a patch of decomposed organic matter) and artificially (a biosolids-amended soil).

It is unknown how the dose of biosolids affects the growth and elemental composition of this species. This information is critical to determine a biosolids application rate that is likely to elicit a significant growth response in *L. scoparium* without resulting in phytotoxicity. Therefore, we aimed to first elucidate the response of *L. scoparium* to increasing doses of biosolids and thence determine the root behavior in a reconstructed soil profile amended with biosolids both homo- and heterogeneously. In the absence of a chemical gradient in the soil, root growth toward a patch of biosolids would be consistent with a signaling mechanism.

## Materials and Methods

### Collection and Preparation of Soil, Biosolids, and Plants

Craigieburn silt loam, a Typic Allophanic Brown Soil (Hewitt, 2010), was collected from Coleridge-Lyndon Rd, Canterbury, New Zealand (S 43° 20′35″, E 171° 36′59″). This area has no history of cultivation nor received any fertilizer. The vegetation is dominated by *Dracophyllum longifolium*, *L. scoparium*, and *Kunzea robusta*. Approximately 100 kg was collected from each horizon (Ah, 0–15 cm; Bw, 20–40 cm; and BC, 40–70 cm), from an area of approx. 25 m^2^, using a spade, and stored separately. Samples were transported to the greenhouse facilities in Lincoln University, NZ (S 43° 38′43″, E 172° 27′44″), and processed in the shade at room temperature, within a week since collection. Samples from each horizon were homogenized and passed through a 12 mm sieve to remove stones while maintaining soil aggregates and structure. A mixture of samples from Ah and Bw horizons in a 4:1 proportion was used in the pot experiment. Subsamples from each horizon, as well as the mixture for pot experiment, were taken for chemical analyses. The Christchurch City Council supplied biosolids from municipal wastewater treatment plant, which were anaerobically digested and thermally dried, and presented as granules. Table 1 shows the chemical properties of the soils and biosolids.

**Table 1:** Chemical characterization of soil and biosolids in the rhizobox and pot experiment.

Parameter	Horizon Ah	Horizon Bw	Horizon BC	Soil for pots	Biosolids
pH	5.57 ± 0.00	5.77 ± 0.04	5.89 ± 0.06	5.56 ± 0.01	6.78 ± 0.02
EC (μS cm^−1^)	36 ± 1.0	17 ± 0.7	7.9 ± 0.3	36 ± 1.0	2690 ± 32
C (%)	1.46 ± 0.18	1.03 ± 0.07	0.70 ± 0.05	1.59 ± 0.04	30 ± 0.03
N (%)	0.24 ± 0.00	0.12 ± 0.00	0.07 ± 0.00	0.22 ± 0.01	3.95 ± 0.00
NH_4_^+^ -N	<0.01	<0.01	<0.01	<0.01	2375 ± 14
NO_3_^−^ -N	0.04 ± 0.02	0.29 ± 0.04	0.17 ± 0.03	<0.01	3.56 ± 0.23
Olsen P	15 ± 0.37	15 ± 0.37	6.2 ± 0.23	18 ± 1.1	506 ± 5.9
S (T)	380 ± 8.5	270 ± 3.5	230 ± 3.3	380 ± 2.4	14000 ± 90
S (E)	<0.01	<0.01	<0.01	<0.01	1807 ± 21
K (T)	3430 ± 88	3750 ± 112	4200 ± 85	3510 ± 51	2160 ± 19
K (E)	87 ± 12	36 ± 9	23 ± 6	102 ± 15	879 ± 40
Ca (T)	4980 ± 96	3730 ± 98	4220 ± 36	4840 ± 58	30500 ± 220
Mg (T)	5570 ± 60	5650 ± 109	6040 ± 52	5620 ± 30	5020 ± 24
Mg (E)	134 ± 14	57 ± 12	9.1 ± 1.8	163 ± 14	916 ± 9
Na (T)	210 ± 7	220 ± 8.4	210 ± 3.7	210 ± 8.4	650 ± 5.9
Na (E)	13 ± 2	18 ± 4.2	8.2 ± 1.9	15 ± 2.9	564 ± 15
Mn (T)	520 ± 2	440 ± 6.3	340 ± 6.2	510 ± 1.9	410 ± 2.2
Mn (E)	4.8 ± 0.6	1.2 ± 0.5	0.4 ± 0.0	4.1 ± 0.8	11 ± 0.3
Cu (T)	8.5 ± 0.22	8.5 ± 0.26	10.7 ± 0.14	8.4 ± 0.1	291 ± 2.4
Cu (E)	<0.01	<0.01	<0.01	0.02 ± 0.00	2.8 ± 0.06
Zn (T)	90 ± 1.3	104 ± 2.6	79 ± 0.7	94 ± 1.4	993 ± 1.8
Zn (E)	0.13 ± 0.05	0.02 ± 0.01	0.06 ± 0.04	0.22 ± 0.07	5.1 ± 0.54
Cd (T)	0.3 ± 0.03	0.29 ± 0.03	0.15 ± 0.04	0.32 ± 0.03	1.6 ± 0.01
Pb (T)	26 ± 0.6	25 ± 0.6	20 ± 0.6	26 ± 0.6	54 ± 0.6

*Average ± standard errors (n = 5). Units are mg kg^−1^ unless otherwise indicated. T, total; E, exchangeable.*

Approximately 100 *L. scoparium* seedlings were obtained in one seedling tray from Department of Conservation of New Zealand Nursery, Motukarara (S 43° 43′39″, E 172° 35′03″). The seedlings of similar size were chosen for both trials, ranging in above ground size from 4 to 6 cm, and having small roots, where the main root was of similar size to the above ground plant and had few laterals (see Supplementary Material). All the seedlings were planted in both experiments within 10 days, to avoid size or age effects prior to the exposure to treatments. Following 4 months of growth, harvesting occurred within 2 weeks for both experiments, and the slow growers (controls) were harvested at the end. The difference in planting and harvesting times was insignificant compared with the length of the experiment.

*Leptospermum scoparium* has mycorrhizal associations (Wicaksono et al., 2017) which would have been present in our soil since *L. scoparium* was initially present. We did not quantify mycorrhizae fungi in these experiments.

### Rhizobox Experiment Setup, Monitoring, and Harvesting

The rhizoboxes used in this work were adapted from the design initially proposed by Wenzel et al. (2001). Here, the rhizoboxes were made with a wooden frame and two glass sides, with inner dimensions of 80 cm × 80 cm × 2.5 cm. The size of rhizoboxes was larger than all other studies (Wenzel et al., 2001; Bott et al., 2008; Reis et al., 2017) to incorporate the development of the plants beyond the small-seedling phase. They were placed on a 30° angle to induce the plant roots to grow along one of the transparent sides, thus allowing real-time observation of the root growth dynamics, as well as eventual targeted sampling of the rhizosphere and bulk soil.

Soil from the three horizons was packed in three layers, each 25 cm deep. A 2 cm layer of approx. 1.5 cm diameter pea gravel was set in the bottom to enhance the drainage. Rhizoboxes were filled horizontally to achieve uniform, but unique, bulk densities within horizons (of 0.88 g cm^−3^ for Ah horizon, 1.12 g cm^−3^ for Bw horizon and 1.30 g cm^−3^ for BC horizon).

Biosolids were applied at a rate of 150 g per rhizobox (approx. 4% w/w of the Ah horizon), resulting in total N addition of 5.93 g, Olsen P addition of 76 mg, and exchangeable K addition of 132 mg. Four treatments with three replicates per treatment comprised: (C) control soil without biosolids, (T) surface-applied biosolids without any incorporation, (M) biosolids mixed homogeneously in the Ah horizon, and (P) biosolids concentrated on one side of the rhizobox in a section occupying one third of the Ah horizon volume. The setup of the rhizoboxes is shown in the Supplementary Material.

*Leptospermum scoparium* seedlings were carefully transplanted from the seedling tray to the rhizobox after having all the potting mixture removed from the roots. Black plastic was placed around the rhizoboxes to prevent light and biofilm growth at the soil-rhizobox interfaces. They were also covered with aluminum foil to avoid a temperature increase from exposure to sunlight. The 12 rhizoboxes were placed randomly and maintained in the greenhouse for 4 months during the NZ summer (December 2015 to March 2016). They were watered daily to field capacity to avoid water stress over the summer, and to avoid possible root foraging behavior toward water instead of nutrients. The mean temperature during the experiment period was 20.2°C with maximum temperature of 32°C and minimum of 9.8°C. The length of the longest shoot was recorded monthly, henceforth referred to as shoot length. The visible roots at the soil-rhizobox interface were mapped by tracing them onto an acetate film, which was subsequently scanned with 400 dpi resolution.

At the end of the experiment, when the roots on the fastest growing treatment (top and mixed application of biosolids) reached the edges of the rhizobox (after 4 months), the shoots were abscised, rinsed with deionized water, and oven-dried at 70°C for 1 week. Once dried, leaves were separated from the stems, bulked together on an individual plant basis and treated separately. For the root collection, the soil material (soil and roots) from each rhizobox was divided into nine equally sized sections. Sections were labeled according to their respective horizon (A, B, or C), and the vertical column from left to right (1, 2, or 3). The roots were then collected from each sector, washed with tap water using a sieve of 1 mm to avoid losing thin roots, and over a container to recover thinner roots that might have passed through the sieve. For each of the sections, the roots were bulked together, oven-dried at 70°C for 1 week, and weighed.

### Pot Experiment Setup, Monitoring, and Harvesting

The pot experiment used 4 L pots (195 mm × 195 mm) with 3.2 kg of soil, and comprised seven treatments and five replicates per treatment. There was a control treatment without biosolids, three treatments with biosolids homogeneously incorporated into the soil at rates of 1.4, 4.3, and 12.8% fresh weight (approx. 16, 48, and 145 t/ha respectively), and biosolids applied on the surface at the same rate. Along the text, the three rates of application are referred to as “low,” “medium,” and “high.” One *L. scoparium* seedling was planted in each pot. The 35 pots were placed in a randomized block design in the same greenhouse as the rhizoboxes above. The trial ran during the same period and the pots were maintained in an identical fashion as the rhizoboxes. The growth of the plants was recorded by measuring the shoot length as before.

At the end of the experiment, shoots were abscised and treated as above. The top 3 cm of the growing medium in the pot (consisting of soil, biosolids, and root material) was separated from the remaining material in the pot below (approximately 11 cm). The roots were treated as above.

### Chemical Analysis

Soluble NH_4_^+^ and NO_3_^−^ concentrations in soils and biosolids were determined on fresh samples, using a 2M KCl extraction (Clough et al., 2001), and analyzed with a Flow Injection Analyser (FOSS FIAstar 5000). Sub samples of soil, and biosolids were dried at room temperature and analyzed for pH and electrical conductivity in a 1:5 (w:v) soil-water ratio (Blakemore et al., 1987). Pseudo-total elements of the soil, and biosolids were extracted using the microwave CEM MARS Xpress acid digest technique (0.5 g substrate, 4.0 mL trace element grade nitric acid and 4.0 mL 30% hydrogen peroxide, according to the equipment specifications). The exchangeable trace element fraction was extracted with 0.05M Ca(NO_3_)_2_ (McLaren et al., 2005). The Olsen P was extracted with 0.5M NaHCO3 (Olsen et al., 1954).

*Leptospermum scoparium* leaves were analyzed for total N using an Elementar Vario-Max CN Analyser. Dried subsamples were digested using a microwave acid digestion technique (CEM MARS Xpress, using 0.3 g dried plant material, 2.0 mL trace element grade nitric acid and 2.0 mL analytical grade 30% hydrogen peroxide, according to the equipment specifications). Due to the small amount of leaves in the control treatments of the rhizobox experiment, the minimum weight required for N analysis was not reached. Therefore, the rhizobox trial did not have the N analyzed in the control treatment.

Concentrations of P, K, S, Mg, Ca, Cr, Mn, Fe, Cu, Zn, As, and Cd were determined in the digests and extracts using inductively coupled plasma optical emission spectroscopy (ICP–OES, Varian 720-ES). Analysis of Certified Reference Materials (reference 981, sandy soil from Netherlands) and a reference plant sample (reference 952, mixture of grasses from Netherlands) from Wageningen Evaluating Programs for Analytical Laboratories (WEPAL, NL-6700 EC Wageningen, Netherlands) gave recoveries between 85 and 120%.

### Data Analysis

Root drawings were analyzed for total root length by WinRHIZO^TM^ software ^1^. To analyze the distribution of roots in the rhizobox, root drawings were divided in 20 cm × 20 cm squares, placing the stem in the top-middle part of one of the squares, and root length in each squared was measured. Note that this division in squares is not the same than the division of soil and root biomass. This is for increasing the resolution of the distribution of root length compared with biomass and soil.

The results of each experiment were analyzed separately. For the rhizobox experiment, total root length, shoot length, plant dry weight, percentage of total biomass allocated to the roots, and concentration of elements were compared between treatments with analysis of variance (ANOVA) and Turkey’s test post hoc multiple comparison testing (p < 0.05). Raw data was tested for homoscedasticity with Bartlett’s test, and for normality with Ryan-Joiner test’s (which is similar to Shapiro-Wilk). Data was Log10 transformed when the assumptions were not fulfilled. Root length at right and left sides of the main root in the Patch treatment were compared with one way paired t-test, and assumption of normality for the differences between both sides with Ryan-Joiner test. Calculations were performed using MINITAB^®^ Release 12.

For the pot experiment, the same responses were analyzed with two-way ANOVA with Block and Treatment as factors. Turkey’s multiple comparison test was used to identify significant differences between treatments. Raw data was tested for homoscedasticity with Bartlett’s test, and for normality with Shapiro-Wilk’s test. When the assumptions were not fulfilled, data was log10 or root square transformed. Calculations were performed using R software.

The few outliers identified during the analysis of the results were removed from the analysis. This is highlighted in the tables and graphics of results by lower number of replicates analyzed. All the graphics were created with Microsoft Excel.

## Results

### Root and Shoot Development

Root and shoot length in the end of the experiment were correlated with root and aerial part biomass. The Person correlation coefficients were 0.955 (*p* < 0.001) for the roots in the rhizobox experiment, and 0.636 (*p* < 0.001) for the whole set of aerial parts in both experiments. Therefore, root and shoot length represent plant growth throughout the experiment.

**FIGURE 1:**
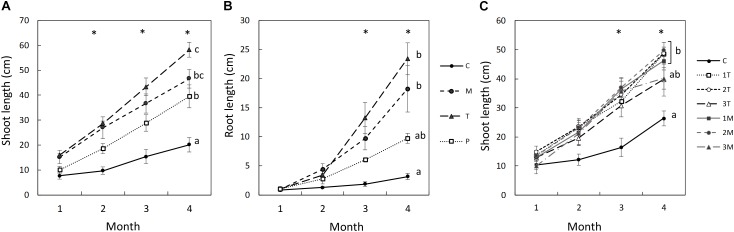
Averages and standard errors of shoot length **(A)** and root length **(B)** of plants throughout the rhizobox experiment, and shoot length in the pot experiment **(B)**. ^∗^ indicates *p* < 0.005 in the ANOVA analysis. Different letters indicate significant differences in total root length between treatments (*p* < 0.05, Turkey’s test), *n* = 3 in **(A,B)**, except for T in **(B)**, with *n* = 2, and *n* = 5 in **(C)**.

In the rhizobox experiment, Mixed (M), and Top (T) treatments were the ones with highest root and shoot length, followed by Patch (P). Control (C) treatment presented significant lower root and shoot growth. In the first month, the total root length, and shoot length were not significantly different between treatments (Figure 1A,B). The roots of C and P treatments were thin with few lateral roots, and the growth was mostly vertical. In contrast, the roots in M and T treatments were thicker with more lateral roots and mainly located top horizon (see Supplementary Material).

From the second month, the shoot length was significantly higher in M and T treatments than in C. And from the third month, both shoot and root length were significantly higher in all treatment with biosolids (M, T, and P) than in C (Figure 1A,B). Shoots in P treatment were not significant different from the control until the third month. From the second month, when the roots in P treatment found the patch of biosolids (see description below), the shoot growth rate of P, 10 cm/month, was similar than M treatment, compared with 4 cm/month in C treatment (Figure 1A).

Similarly, in the pot experiment, the shoot lengths in *L. scoparium* were significantly higher in the biosolids treatments compared with the control (Figure 1C). Although there was a significant difference in the third month, by the fourth month, the treatments with the high rate of biosolids were not different from the control. There were no significant differences between the type of application T or M.

**FIGURE 2:**
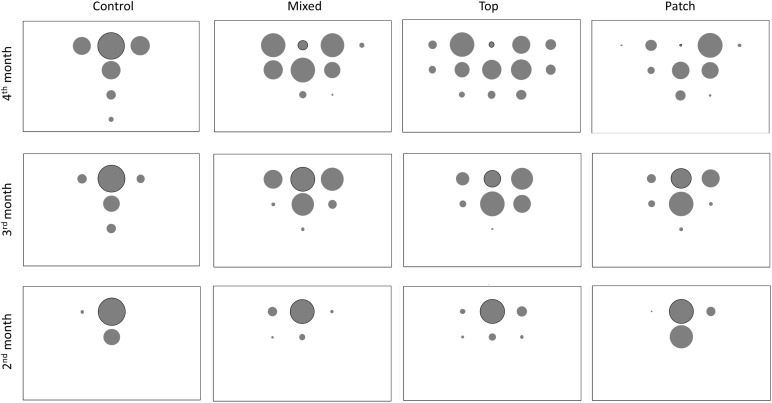
Distribution of new roots in each 20 cm ×20 cm quadrants in each treatment. Bubble size (area) is proportional to the average % of new roots in each quadrant. Outlined bubble indicates the quadrat where the main root was *N* = 3 except for T where *N* = 2.

**FIGURE 3:**
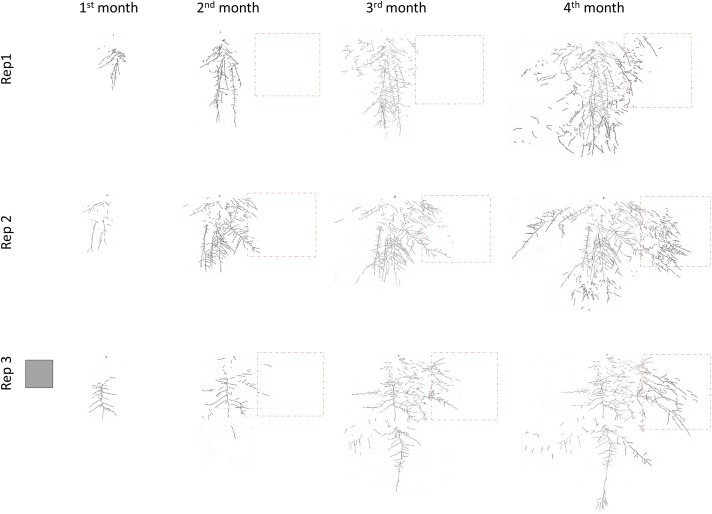
Development of *L. scoparium* roots growing in the three replicates of patch (P) treatment in the rhizobox experiment. The gray square represents the scale of 10 cm × 10 cm. The orange square represents the area where the patch of biosolids was located.

Figure 2 shows the average percentage of new roots generated in each quadrant in each treatment and month. Data of the percentages with standard error are in Supplementary Material. The roots in M treatment developed mostly in the Ah horizon and were less spread than in the T treatment (Figure 2 and Supplementary Material). At the end of the experiment, 95% and 88% of the root biomass in M and T, respectively was located in the Ah horizon. The total root length in C treatment throughout the experiment was significantly lower than in M and T treatments, but roots developed more vertically (Figure 1B, 2, 3). Figure 3 represents all the root systems of plants in P treatments along the experiment. Roots in P treatment started to grow preferentially in/or toward the patch of biosolids from the second month (Figure 3), increasing the number of lateral roots at right of the main root (in direction of the patch), and also increasing density of thin roots (more branching) toward, and in the patch. In all p-treatment rhizoboxes, the length of the new roots length was consistently higher to the right of the main root (toward the patch) than on left of the main root (Figure 2), leading to an average new root length on the right hand side that was significantly higher than the left hand side at the end of the experiment.

**FIGURE 4:**
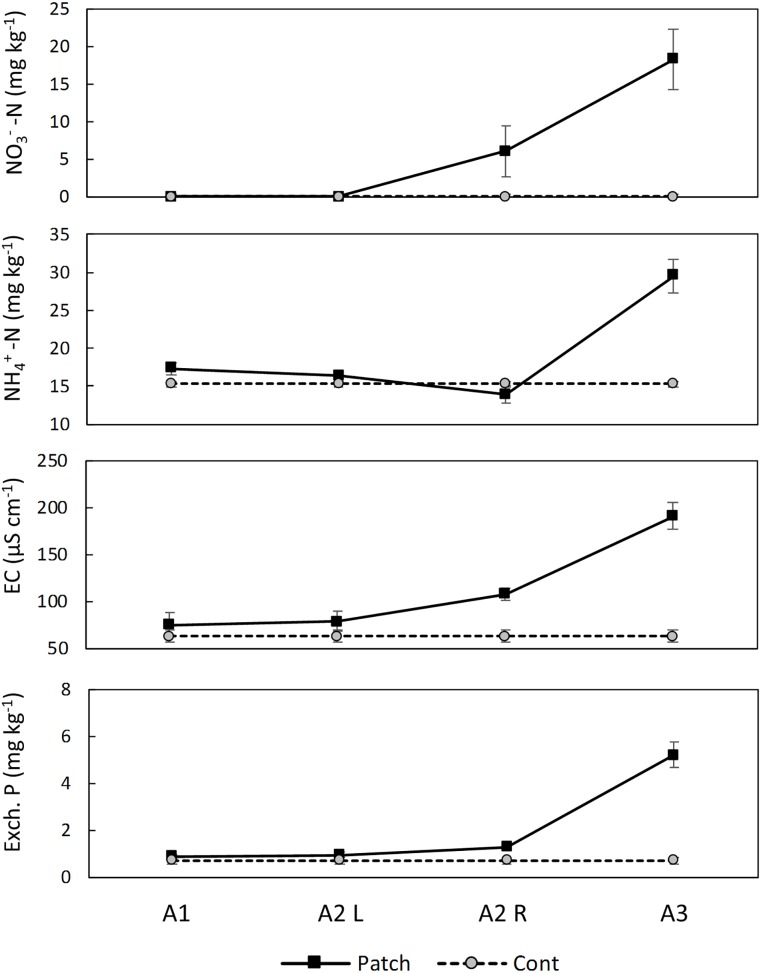
Soil analysis in the Ah horizon of the rhizoboxes at the end of the experiment. Quadrat A2 was divided to be able to detect a gradient. A3 is the quadrat where the patch of biosolids was located. Results represent average and standard errors (*n* = 6). EC, electrical conductivity.

The analysis of the soil in the rhizoboxes indicated horizontal gradients of NO_3_^−^ and EC from the patch of biosolids, but not for extractable NH_4_^+^, or P (Figure 4).

### Plant Biomass

**FIGURE 5:**
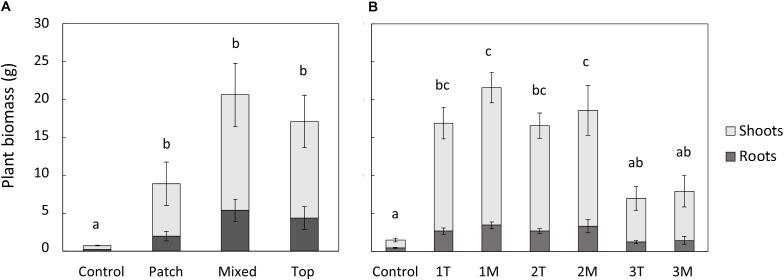
Plant biomass (shoots and roots) in the rhizobox **(A)** and pot **(B)** experiments. In **(B)** 1, 2, and 3 are the increasing doses of biosolids, T is top application, and M is mixed application. Bars represent standard error. Different letters indicate significant differences in the total plant biomass (root+shoot) (*p* < 0.05, Turkey’s test). *N* = 3 in **(A)** and *n* = 5 in **(B)**.

The plant biomass (shoots and roots) obtained for the treatments in the rhizobox experiment followed the sequence M = T = P > C (Figure 5), similar to the results obtained for plant development (shoot and root length), and the enhanced growth resulting from biosolids application. No significant differences between treatments could be observed in the percentage of the total biomass allocated to roots.

**FIGURE 6:**
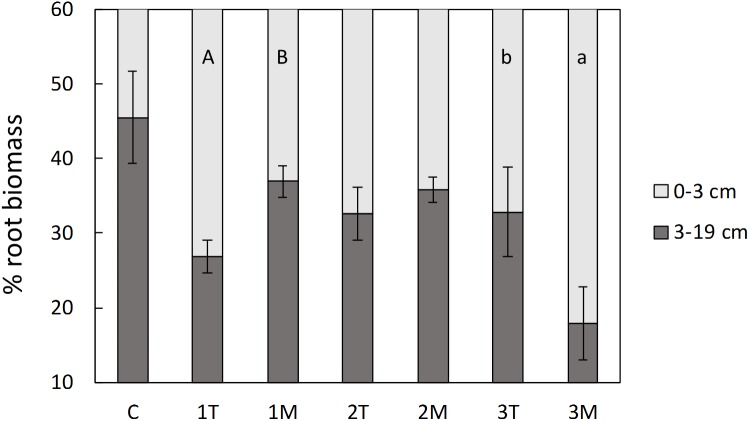
Distribution of root biomass in the top or bottom part of the pots. C, control; 1, 2, 3, increasing dose of biosolids; T, top application; M, mixed application. The error bars represent standard error. Different letters indicate significant difference between top and mixed application of biosolids at the same rate, capital letters for rate 1, small letters for rate 3 (*p* < 0.05, *t*-test).

In the pot experiment, both shoot and root biomass, were significantly higher than the control in the treatments with low and medium rate of biosolids application (16 and 48 t/ha, respectively), but no significant difference was observed between type of application (T or M; Figure 5). The high dose of biosolids (145 t/ha) in both types of application (T and M) had a negative effect in the biomass of the plants (Figure 5), compared with low and medium rates, and was not significantly different from control. This was also visually evident with less vigor and foliage density (see Supplementary Material), as well as small necrosis spots on the leaves. The percentage of the total biomass allocated to roots was significantly higher in control treatment (average of 32%) than in all biosolids treatments (average of 18%). There were no significant differences between biosolids treatments, rate or type of application (results not shown). Although the type of application (T or M) did not affect the root biomass, the distribution of root biomass in the pot was different depending on type of application, as shown in Figure 6. In the low dose treatment, the percentage of root biomass in the top 3 cm was significantly higher when biosolids were applied in the top than when they were mixed. At medium rate, the root distribution was unaffected by the type of biosolids application. At the highest dose, there was a higher concentration of roots in the surface in mixed application of biosolids, compared with top application.

### Plant Nutrition and Trace Elements

In the rhizobox experiment, the N concentration in leaves was similar in all treatments with different types of biosolids application (M, T or P – there are no leaf N results for C) (Table 2). In the pot experiment, the concentration of N in leaves of *L. scoparium* increased in the treatments with biosolids compared with control, although it was only significant between control and 1M vs. 3M. Nitrogen is supplied by biosolids almost entirely as NH_4_^+^ (Table 1); however, there is some evidence to suggest that a proportion of this was converted to the more mobile NO_3_^−^ during the growth period (Figure 4).

**Table 2:** Average nutrient and trace element concentration in *L. scoparium* leaves in both experiments.

Treat.	N % w/w		P g kg^−1^		S g kg^−1^		K g kg^−1^		Mg g kg^−1^		Ca % w/w		Na g kg^−1^	Cu mg kg^−1^		Mn mg kg^−1^		Zn mg kg^−1^		Cd mg kg^−1^	
**Rhixobox experiment**																					
ANOVA			0.000						0.000^#^		0.002			0.033^#^		0.001^#^		0.040			
Control	n.a.		2.5	b	3.0		8.2		4.7	b	1.8	b	1.8	7.1	b	185	a	41		0.00	
Mixed	2.3		1.4	a	2.7		8.0		2.6	a	1.5	b	0.85	5.2	ab	397	b	42		0.03	
Patch	2.3		2.3	b	2.9		8.0		2.5	a	1.0	a	1.0	5.6	ab	255	a	29		0.04	
Top	2.2		1.0	a	2.4		6.7		2.0	a	1.0	a	1.0	4.4	a	403	b	21		0.01	

**Pot experiment**																					
ANOVA	0.005^∗^		0.001		0.005		0.022		0.000^#^		0.000^#^			0.005		0.000^#^		0.000^#^		0.000^&^	
Control	2.0	a	1.4	ab	3.2	ab	12	b	4.1	cd	1.4	bcd	1.1	6.3	b	292	a	61	ab	0.02	ab
1T	2.4	ab	1.5	abc	3.1	ab	8.9	ab	1.9	a	0.89	a	1.0	5.3	ab	267	a	36	a	0.01	a
1M	2.2	a	1.2	a	2.5	a	8.5	ab	2.1	ab	0.97	ab	0.72	3.2	a	241	a	40	a	0.01	a
2T	2.6	ab	2.0	bc	3.0	ab	9.0	ab	2.5	ab	1.2	abc	0.85	4.3	ab	720	bc	42	a	0.13	bc
2M	2.7	ab	1.9	abc	3.4	b	9.1	ab	2.6	b	1.4	cd	1.0	5.5	b	578	b	62	ab	0.12	abcd
3T	2.7	ab	1.8	abc	2.9	ab	6.1	a	2.6	b	1.1	abc	0.85	3.7	ab	803	bc	41	a	0.15	cd
3M	2.8	b	2.1	c	3.5	b	6.0	a	3.7	d	1.7	d	0.85	5.6	ab	977	c	88	b	0.22	cd

*ANOVA, indicates the p-value of the ANOVA analysis in each experiment for each element. Different letters indicate significant differences (p < 0.05, Turkey’s test) between treatments in the same experiment. Where no ANOVA or letters are present indicates that there were no significant differences. ^∗^None of the transformations fulfilled the assumptions, ANOVA made in raw data. ^#^Log10 transformation. ^&^Square root transformation.*

Phosphorus concentrations in *L. scoparium* leaves in the rhizobox experiment were higher in C and P treatments than in T and M treatments (Table 2). In the pot experiment, P concentration increased with higher rates of biosolids application, and was not different in surface application compared with mixed application (Table 2). Olsen P was 30 times higher in biosolids than in the soil (Table 1).

Although exchangeable S, K, Mg, Ca, and Na were higher in biosolids than in the soil (Table 1), the concentration of these elements in leaves were not higher in biosolids treatments than in control, because they were not limiting nutrients in the soil. However, there are differences in the concentration of S, Mg, and Ca between doses of biosolids (Table 2).

The nutrient accumulation by leaves (concentration × leaf biomass) was mainly affected by the differences in biomass, rather than by nutrient concentration, being always higher, for all the nutrients, in treatments with low and medium biosolids application in the pot experiment, and M and T in the rhizobox experiment (see Supplementary Material).

In the rhizobox experiment, both Cu and Zn concentrations in the leaves of the plants treated with biosolids were low and similar to the values obtained in the control treatment (Table 2). Despite high concentrations, especially of Zn, in biosolids, the bioavailability of this element for plants was low or maybe was retained at the root level. Manganese concentrations were higher in M and T treatments than in C and P, while no significant differences were found for leaf concentrations of Cd.

In the pot experiment, the leaf concentration of trace elements increased as the application doses of biosolids increased (Table 2). Concentrations of Mn and Cd increased significantly from the control, with ten (Cd), and two (Mn) fold increases observed between the low and medium dose treatments. On the other hand, leaf concentrations of Cu and Zn did not increase significantly with increased biosolids application rates. The type of application did not influence the accumulation of trace elements in the leaves except in the case of the high dose of biosolids, and for Zn concentration.

## Discussion

The results were consistent with *L. scoparium* roots having plastic root architecture, with roots foraging and proliferating near and/or into nutrient-rich patches in the soil. *L. scoparium* roots follow a horizontal gradient of NO_3_^−^ until they reach the source in the patch of biosolids. Roots developed more lateral roots and with more branching following the NO_3_^−^ gradient. Due to the existence of a gradient, there is no evidence of a signaling mechanism, although we cannot entirely discard this hypothesis. The preferential allocation of roots in the top of the pots, when biosolids are surface-applied at low doses, is consistent with the observations in the rhizobox experiment. Higher leaching of nutrients to deeper parts of the pots from the surface-applied biosolids at medium rate might explain that roots were not concentrated in the top 3 cm of the pot, and also the extension of roots in the treatment with biosolids applied in the surface in the rhizobox experiment.

The root systems of *L. scoparium* in our rhizoboxes were consistent with the findings of Watson and O’Loughlin (1985) who reported a high proportion of fine roots. Although *L. scoparium* has not been classified according to Grime’s plant strategies (Grime, 2001), its predominance in low fertility soils is consistent with “stress-tolerant” type. However, *L. scoparium* growth and traits are contrary to the traits associated with plants in this category: *L. scoparium* is not a slow-growing species, and our results show high morphological root plasticity.

This foraging behavior may enhance the effectiveness of *L. scoparium* to obtain nutrients in the low-nutrient environments where it usually grows (Stephens et al., 2005). This behavior depends as much on the environment as on the plant species (Hodge, 2009). The efficacy of root foraging in increasing plant growth was lower in the present study than in Reis et al. (2017), because the patch of biosolids was further from the main root, and occupied a lower percentage of the rhizobox surface, compared with Reis et al. (2017). Even so, when roots started to forage the NO_3_^−^ gradient, and/or patch of biosolids, plant growth was similar to the plants which more homogeneous distribution of biosolids. The efficacy of this foraging behavior, and low N requirement are highlighted in one of the replicates in Patch treatment, where roots were proliferating in the NO_3_^−^ gradient, near the patch of biosolids, but not inside (Figure 3, Rep1). This replicate presented a growth and nutrient status similar to the other replicates, and to the other treatments with biosolids.

Although *L. scoparium* naturally grows in low fertility environments (Stephens et al., 2005), its growth is enhanced by the addition of nutrients (Esperschuetz et al., 2017a,b; Reis et al., 2017). In this experiment, N was the limiting nutrient; its concentration in leaves when biosolids were added, was comparable to previous studies with this species (Esperschuetz et al., 2017a,b; Reis et al., 2017), and other native pioneer species (Dickinson et al., 2015; Gutiérrez-Ginés et al., 2017). Although it was not observed in this experiment, phosphorus and sulfur can also be limiting in other soil types (Reis et al., 2017). The nutrient requirement of *L. scoparium* is low. In this experiment, as well as in previous ones, N and P leaf concentration, even when supplemented with biosolids, is low compared with general plant requirements for these nutrients: 1–5% for N, and 0.3–0.5% for P (Marschner, 2012). This explains the lack of response to the medium dose of biosolids (48 t/ha) compared with low dose of biosolids (16 t/ha) in the pot experiment; the nutrient requirements of *L. scoparium* were fulfilled with the lowest dose. In the rhizobox experiment, it explains that the replicate that was not proliferating roots inside the patch of biosolids but next to it (Figure 3, Rep1), had a growth similar to the other two replicates with root proliferation inside the patch of biosolids. The root foraging behavior explains the similar growth and nutrient status of *L. scoparium* regardless the distribution of biosolids in the soil, either applied in the surface, mixed in the soil, or concentrated in a patch.

The reduced growth of *L. scoparium* at the highest rate of biosolids application may be due to one or more factors acting either together, or individually. The application of biosolids increased the soil salinity (Table 1 and Figure 4). Other authors have highlighted the risk of increasing soil salinity with high doses of sewage sludge (Reddy and Crohn, 2012; Pérez-Gimeno et al., 2016), which can cause a depletion in plant survival (Fuentes et al., 2010). *L. scoparium* has been previously considered salt tolerant (Cassaniti et al., 2009). Our experiments revealed no increase in foliar Na in higher doses of biosolids. This indicates that the reduced growth is not caused by Na accumulation, but may be due to water stress (induced by the elevated salinity), toxicity effects, or competition with other nutrients. Although Cl^−^ was not analyzed, its presence is probably linked to the high Na concentration in biosolids. Cl^−^ is usually easily bioaccumulated compared with some cations (Gutiérrez-Ginés et al., 2016), which might be a reason for reduced growth in the high dose of biosolids. The Mn concentration in the leaves were highest in the highest dose treatments: above 400 mg kg^−1^, which can be considered phytotoxic (Chaney, 1989). High Mn concentrations and the toxicity symptoms in leaves (small necrosis spots) could explain this as a reason for toxicity of high doses of biosolids application. Since all the nutrients and elements analyzed were supplied by biosolids in higher concentration than in soil (Table 1), their increase in plants exposed to biosolids cannot be attributed to either their higher concentration in amended soil, or the potential synergies between those elements, such as Ca and Mg, or Cu, Zn, and Mn (Marastoni et al., 2019). The concentration of K, on the contrary, significantly decreased in the high dose treatments (3T and 3M, Table 2), despite its higher concentration in biosolids. The high extractable NH_4_^+^ concentration in the biosolids (Table 1) would explain an induced deficiency in K, especially in the early stages of growth before NH_4_^+^ in the biosolids is nitrified to NO_3_^−^. High NH_4_^+^ concentrations have been linked to reduced plant uptake of K (Maathuis and Sanders, 1996).

*Leptospermum scoparium* roots response to this dose of biosolids reflect an avoidance behavior (as explained by Robinson et al., 2009), locating most of the root biomass in the three first cm of the pot, compared with the rest of the treatments (Figure 6). The concentration of biosolids in the pots with the highest rate of application was 12.4% (w/w). This is the same concentration of the patch of biosolids in the rhizobox experiment, and might explain the response of one of the replicates in the Patch treatment, which was not proliferating its roots inside the patch until the last weeks of the experiment (Figure 3, Rep1). Although that concentration of biosolids was detrimental when the whole root system was exposed to it (like in the pot experiment), their presence as a patch has not produced an increase in foliar Mn, or decrease in foliar K, even in the replicates that allocated a big proportion of their root system inside the patch. This indicates that the patch of biosolids produce mainly positive effects in growth and nutrient status. The proportion of roots that are not in contact with the biosolids might overcome the potential toxicity, or salinity in the patch.

## Conclusion

*Leptospermum scoparium’s* roots foraged biosolids patches in soil with root growth following a NO_3_^−^ gradient, which was the limiting nutrient in the soil. This growth pattern did not require a signaling mechanism. Instead, more lateral roots and with more branching are generated in the direction of the patch, and inside the patch. This adaptation for capturing nutrients, and low nutrient requirement, allows *L. scoparium* to benefit (more growth and better nutrient status) from small increases in nutrient availability in the soil, provided either by small doses of biosolids, or by the NO_3_^−^ gradient produced by a patch of biosolids. Regardless the type of application (in the surface, mixed in the soil, or concentrated in a patch), *L. scoparium* grew better and had higher N concentration in leaves in the presence of biosolids than in control without biosolids. However, high doses of biosolids (∼12% w/w) hinders the growth and vigor of the plants, which may be due to the effect of salinity, Mn toxicity or NH_4_^+^-induced K deficiency acting either together or individually. The roots of *L. scoparium* roots also respond to this stress avoiding the proliferation of roots into areas where these negative effects prevail.

## Data Availability Statement

Datasets are available on request. The raw data (.xls and .jpg documents) supporting the conclusions of this manuscript will be made available by the authors, without undue reservation, to any qualified researcher.

## Author Contributions

MG-G co-designed the experiments, coordinated the experimental methods, analyzed the results, and wrote the manuscript. EM participated in monitoring and harvesting the experiments, analyzing the results, and writing the manuscript. NL participated in the design of the experiments, harvesting the experiments, soil analysis, and writing the manuscript. RM participated in the collection of soils, experimental design, setting up the experiments, and reviewed the manuscript. JH participated in the experimental design and reviewed the manuscript. ND participated in the experimental design and reviewed the manuscript. BR participated in the experimental design, the hypothesis, discussion of results, and writing the manuscript.

## Conflict of Interest Statement

The authors declare that the research was conducted in the absence of any commercial or financial relationships that could be construed as a potential conflict of interest.

## Footnotes

1 www.regent.qc.ca
