# Supplementary material for: Response of a Pioneering Species (Leptospermum scoparium J.R.Forst. & G.Forst.) to Heterogeneity in a Low-Fertility Soil
Source: Front Plant Sci. 2019 Feb 6;10:93. doi: 10.3389/fpls.2019.00093 (PMC6372564; doi:10.3389/fpls.2019.00093)
Supplement: Supplementary file 1 [file Data_Sheet_1.PDF]

Supplementary Material

Response of a pioneering species (*Leptospermum scoparium* J.R.Forst. & G.Forst.) to heterogeneity in a low-fertility soil

Maria Jesus Gutiérrez-Ginés*, Engracia Madejón, Niklas J. Lehto, Roger D. McLenaghen, Jacqui Horswell, Nicholas Dickinson, Brett H. Robinson.

*** Correspondence:** Maria Jesus Gutiérrez-Ginés: maria.gines@esr.cri.nz

1 Supplementary Data

The Supplementary data shows the rhizobox experiment set up, and harvest, results of root development, results of pot experiment, and a table with nutrient uptake.

Supplementary Figure 1. Filling of rhizoboxes with the soil from the three horizons. Patch of biosolids is located in the upper horizon at the right third.

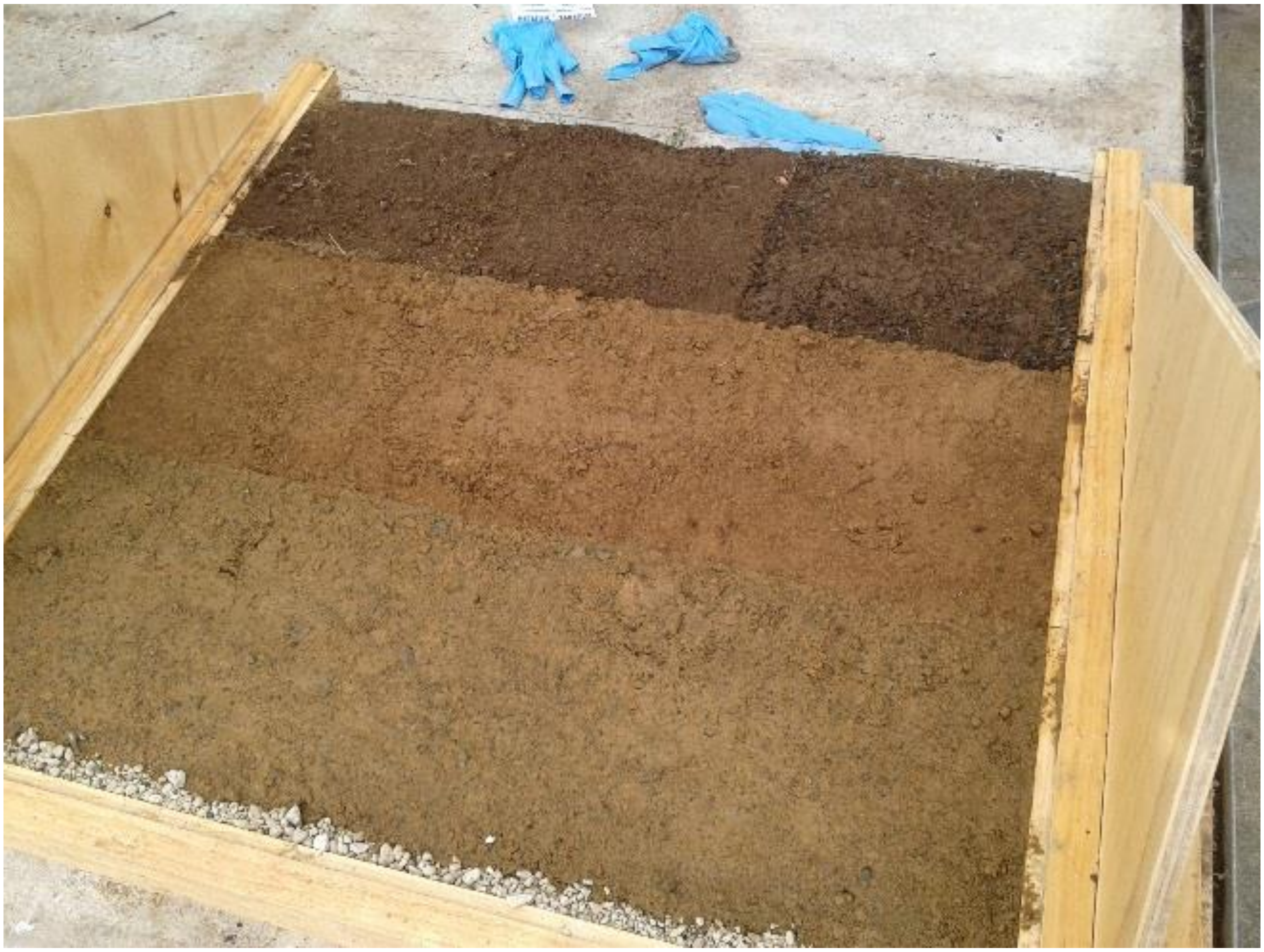

Supplementary Figure 2. *L. scoparium* seedling (4 cm high) when planted in the rhizobox. All the seedlings chosen for both rhizobox and pot trials were similar to the one shown in this photograph.

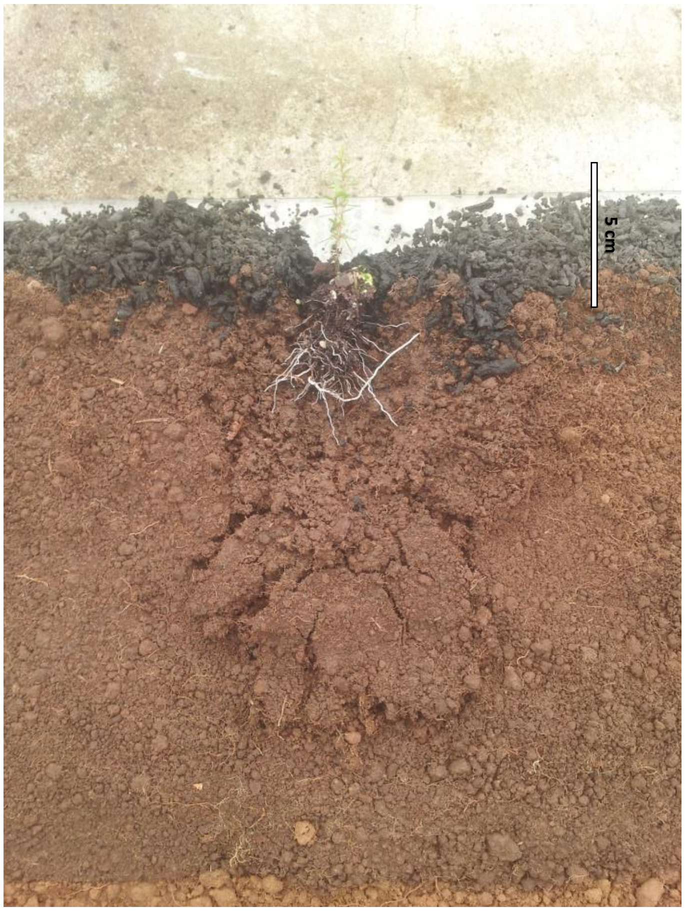

Supplementary Figure 3. Arrangement of rhizoboxes in the greenhouse. Photo at left shows the black plastic covering the rhizoboxes. Photo at right shows the reflecting silvery layer to avoid heating of the soil.

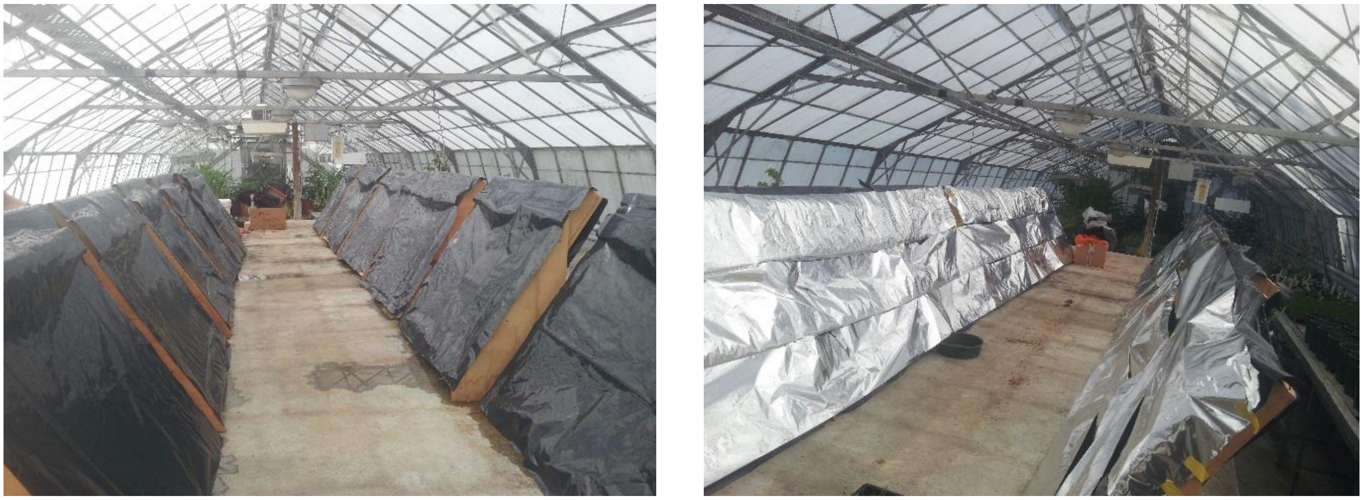

frontiers

Supplementary Figure 4. Example of the 20x20 cm quadrats in which the drawings of the roots were divided for studying the distribution of root length.

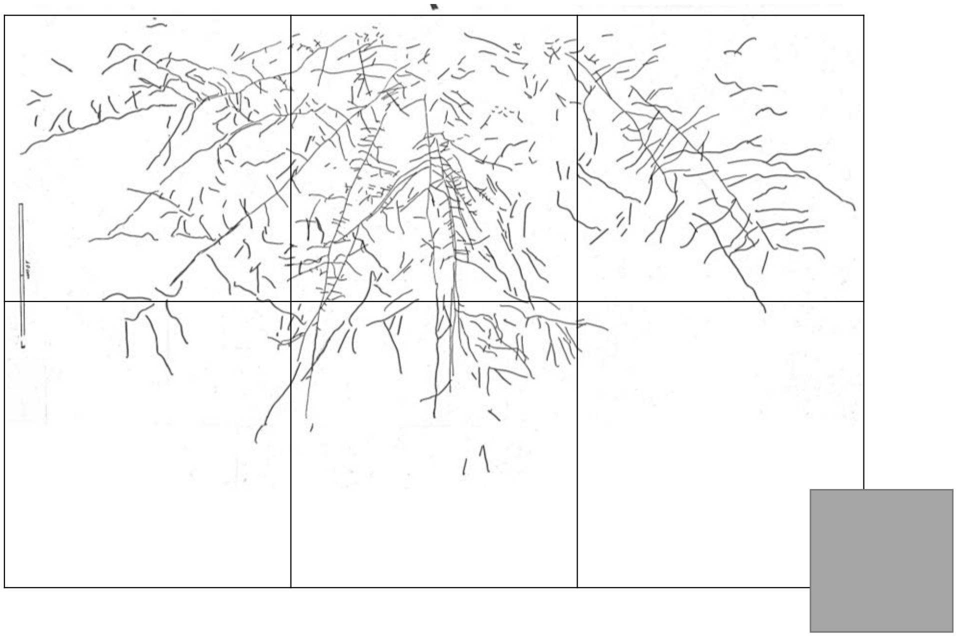

Supplementary Figure 5. Collection of roots in the rhizoboxes in quadrats.

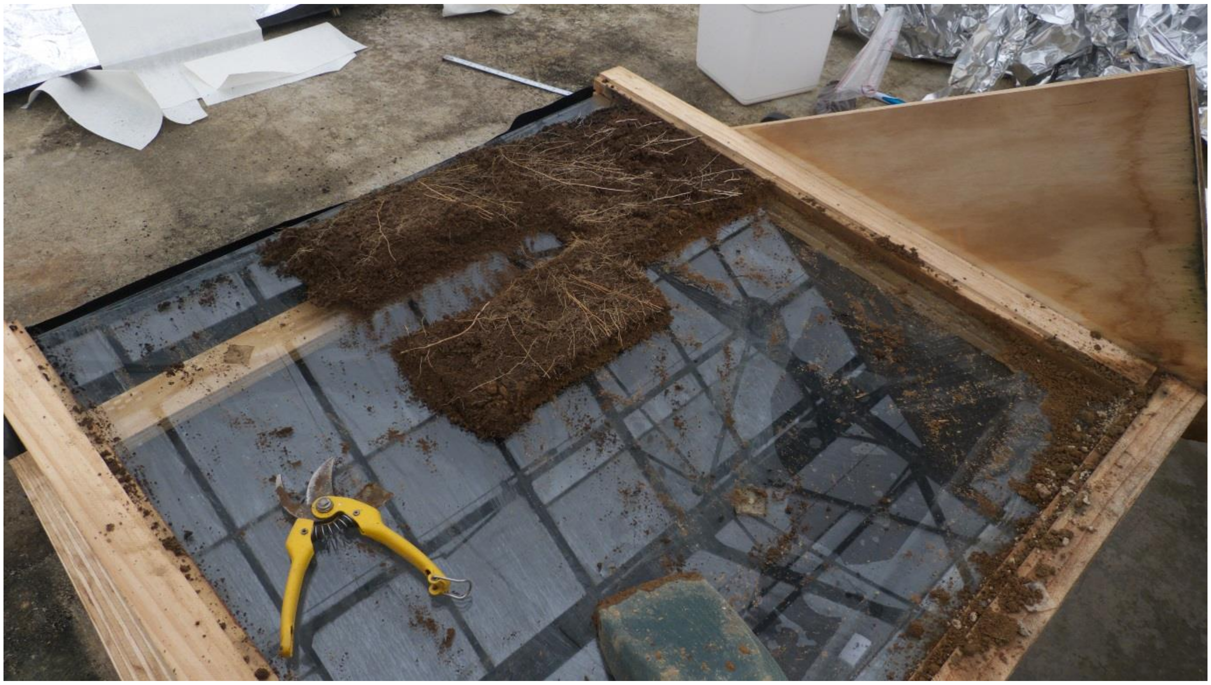

Supplementary Figure 6. *L. scoparium*'s roots in the 12 rhizoboxes after 1 month of growth. The grey square represents a 10 cm x 10 cm square.

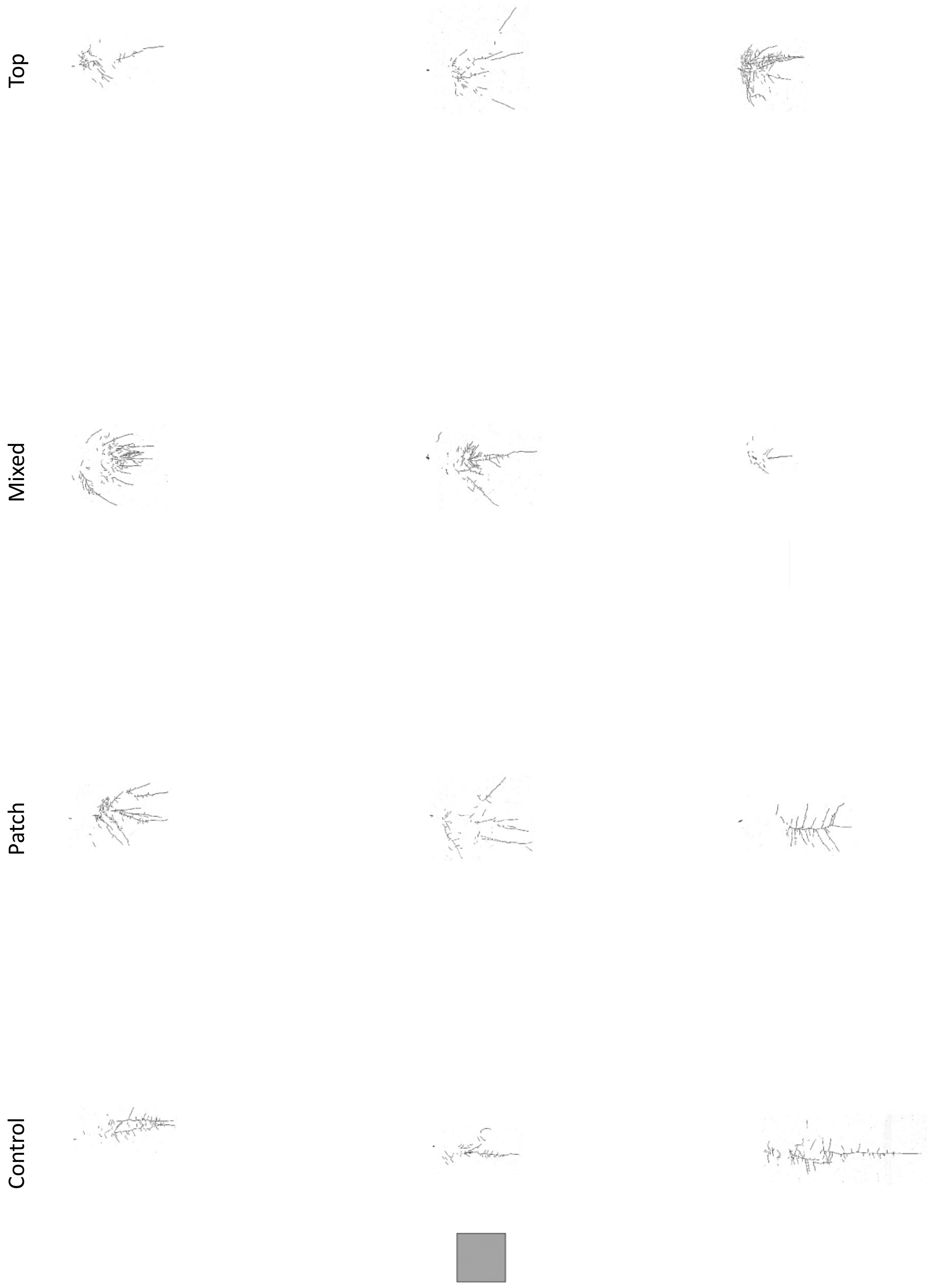

Supplementary Figure 7. *L. scoparium*'s roots in the 12 rhizoboxes after 2 months of growth. The grey square represents a 10 cm x 10 cm square.

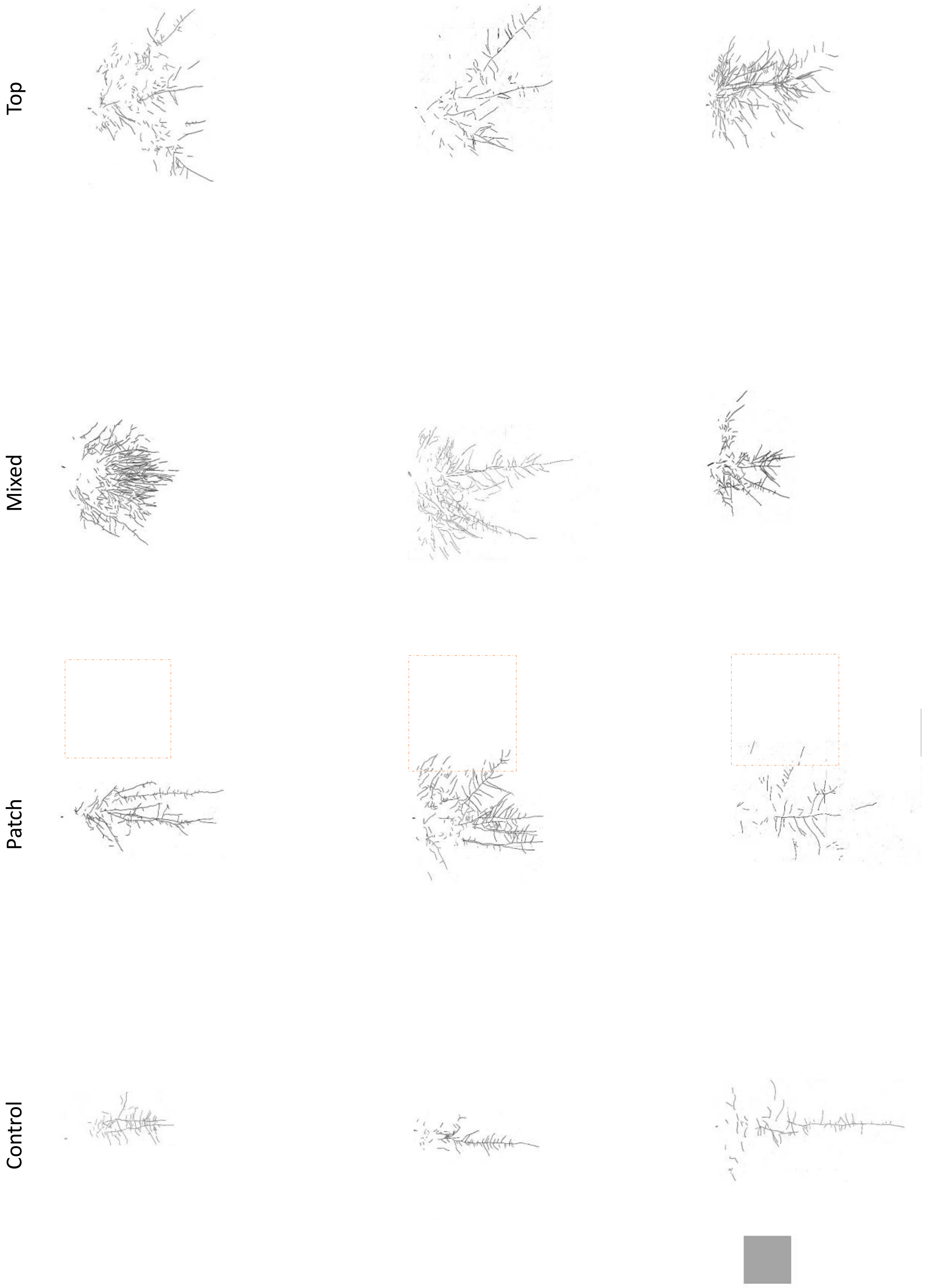

Supplementary Figure 8. *L. scoparium*'s roots in the 12 rhizoboxes after 3 months of growth. The grey square represents a 10 cm x 10 cm square.

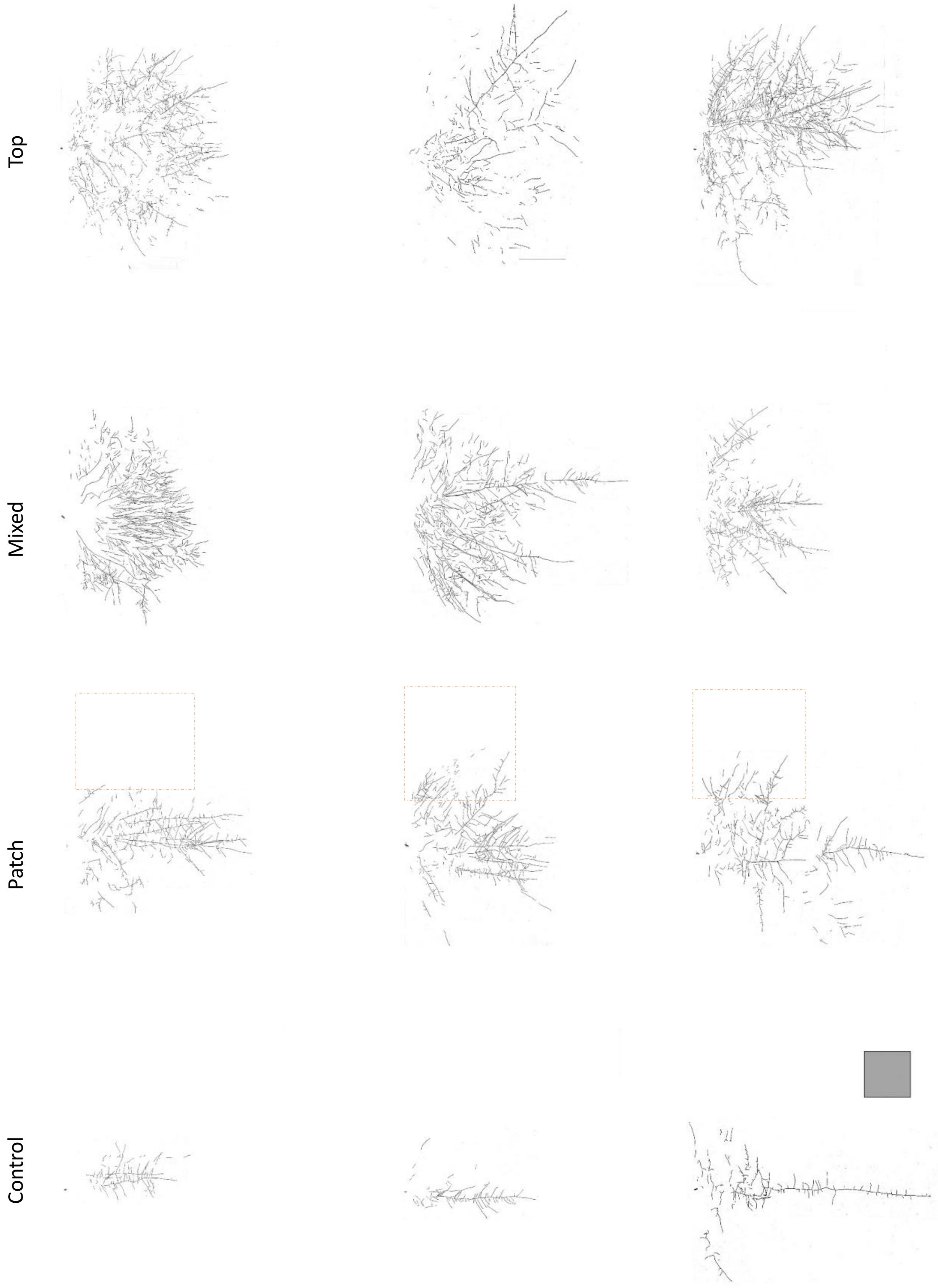

Supplementary Figure 9. *L. scoparium*'s roots in the 12 rhizoboxes after 4 months of growth. The grey square represents a 10 cm x 10 cm square.

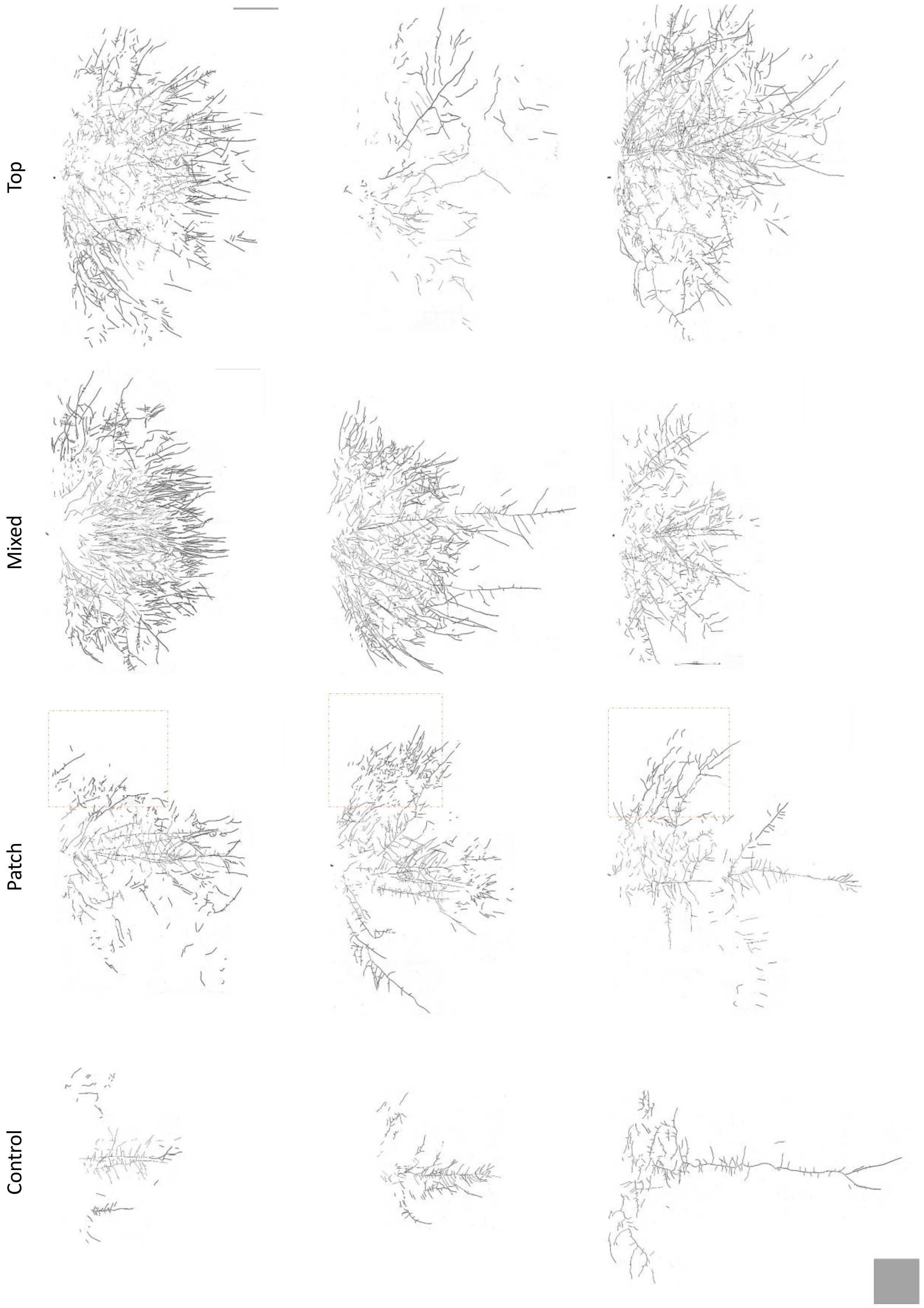

Supplementary Figure 10. *L. scoparium* plants in the end of the pot experiment, exposed to increasing biosolids application. These results correspond to application of biosolids homogenously mixed in the soil. There was not visual difference between these treatments and the ones with surface application with biosolids.

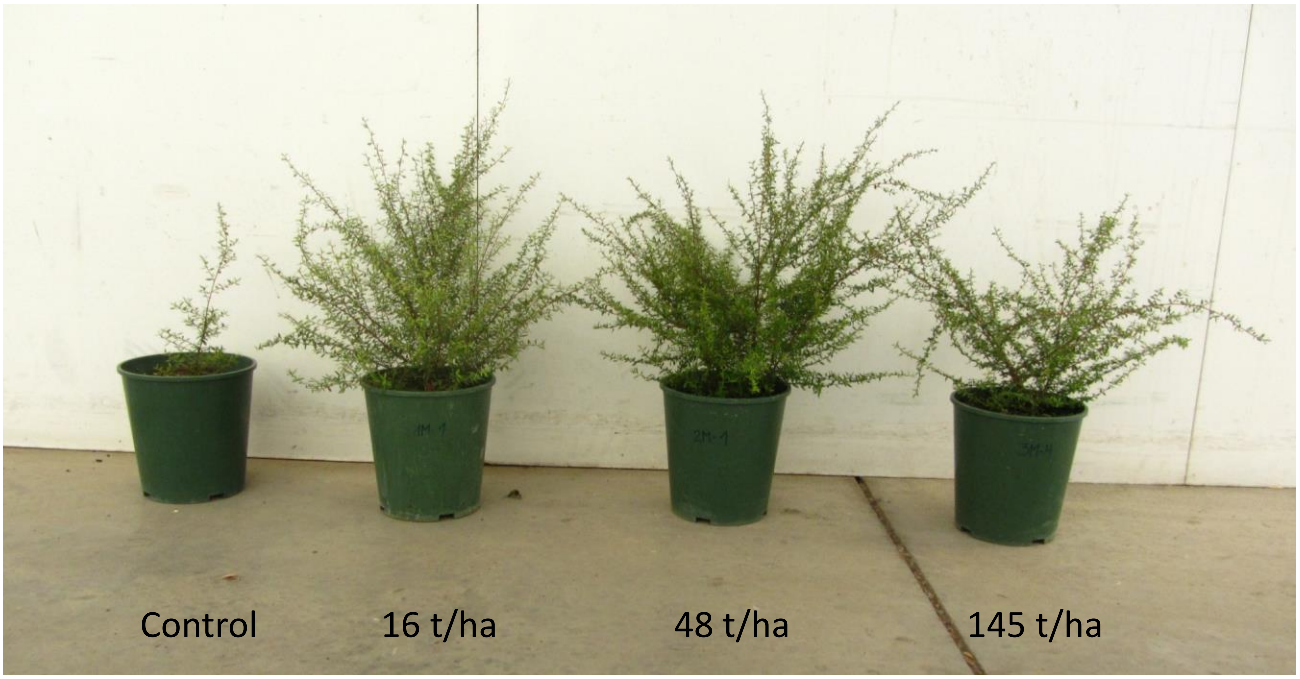

Supplementary Figure 11. Rhizobox with patch of biosolids in the red square at the end of the experiment.

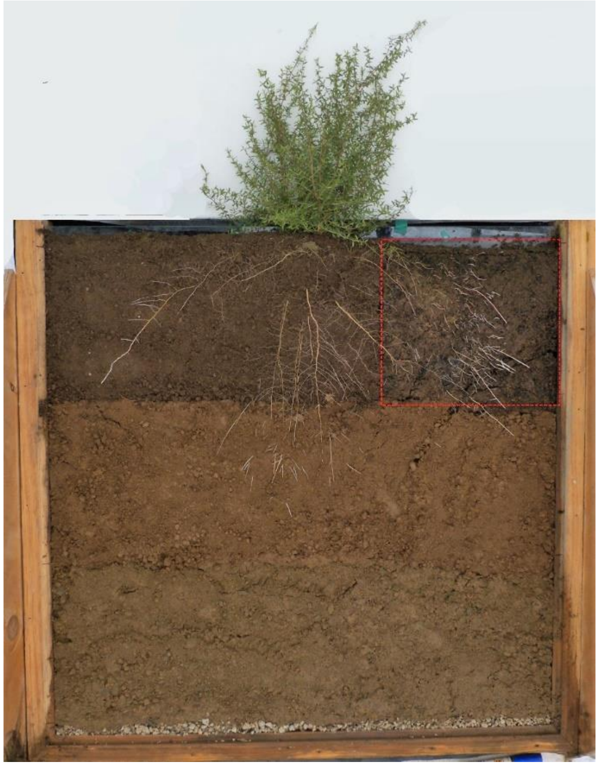

Supplementary Table 1. Average nutrient uptake (mg) by *L. scoparium* leaves in both experiments. Different letters indicate significant differences between treatments in the same experiment.

	N		P		S		K		Mg		Ca	
Rhixobox experiment												
Control			1.3	a	1.5	a	4.2	a	2.4	a	9.2	a
Mixed	361	a	21	b	42	b	116	b	40	b	240	c
Patch	156	a	16	ab	20	ab	58	ab	16	ab	70	ab
Top	282	a	13	ab	30	b	78	b	26	ab	135	bc
Pot experiment												
Control	23	a	1.5	a	3.6	a	14	a	4.6	a	16	a
1T	345	b	21	bc	45	b	124	b	27	bcd	127	bc
2T	364	b	29	c	42	b	124	b	35	cd	168	cd
3T	158	a	10	ab	17	a	32	a	15	ab	61	ab
1M	397	b	21	bc	45	b	153	b	38	cd	173	cd
2M	403	b	29	c	51	b	123	b	40	d	221	d
3M	166	a	13	ab	22	a	41	a	21	abc	102	abc

Supplementary Table 2. Average and standard deviation of the percentage of new roots in each 20 x 20 cm quadrats, n=3 except for Top in months 3 and 4, where n=2. Shaded cell indicates the quadrat where the main root was.

Control

2nd month

1.3 ± 2.3	72.7 ± 30.9	
	26.0 ± 28.6	

3rd month

7.5 ± 10.6	60.3 ± 16.1	5.7 ± 5.6
	22.1 ± 16.6	
	6.9 ± 11.9	

4th month

	17.2 ± 11.4	38.6 ± 21	19.7 ± 14.9	
		18.3 ± 7.7		
		4.8 ± 8.4		
		1.3 ± 2.3		

Mixed

2nd month

12.2 ± 11.7	80.3 ± 17.1	1.6 ± 2.8
0.8 ± 1.3	5.2 ± 6.6	

3rd month

16.9 ± 6.5	28.9 ± 17.9	24.8 ± 2.7
0.7 ± 0.5	24.4 ± 14.3	3.7 ± 5.2
	0.7 ± 1.1	

4th month

	23.4 ± 10.7	4.0 ± 6.9	22.2 ± 4.7	0.9 ± 1.2
	14.8 ± 8.6	23.9 ± 5.1	10.2 ± 9.4	
		1.9 ± 3.2	0.2 ± 0.3	

Top

2nd month

3.3 ± 4.7	74 ± 10.5	13.1 ± 8.1
1.1 ± 1.6	6.8 ± 2.6	1.7 ± 1.4

3rd month

9.2 ± 9.5	14.8 ± 0.8	25.1 ± 12.4
2.3 ± 3.3	31.8 ± 3	16.4 ± 3.8
	0.3 ± 0.4	

4th month

3.0 ± 2.3	24.3 ± 4.9	1.4 ± 1.9	13.1 ± 2.5	4.5 ± 4.0
2.2 ± 0	9 ± 1.2	14.9 ± 5.8	17.4 ± 1.5	4.1 ± 5.6
	1.4 ± 0.0	2.6 ± 1.0	4.1 ± 4.9	

Patch

2nd month

0.3 ± 0.5	49.4 ± 18.7	6.7 ± 5.8
	43.7 ± 22.4	

3rd month

5.2 ± 2.3	27.3 ± 12.8	21.8 ± 14
3.2 ± 3.3	40.5 ± 16.4	1.0 ± 1.8
	0.9 ± 1.6	

4th month

0.2 ± 0.4	8.6 ± 11.2	0.5 ± 0.4	40.3 ± 9.4	0.9 ± 1.0
	3.3 ± 1.8	20.4 ± 10.3	18.8 ± 7.2	
		7.3 ± 6.4	0.5 ± 0.8	

Supplementary Table 3. Results of the two-way ANOVA for the data from the pot experiment. Df, degrees of freedom; F, F-statistic; p, p-value; DW, dry weight; √, square root transformation; $Log_{10}(X)$, Log_{10} transformation

		√ DW		Shoot Height_1		Shoot Height_2		Shoot Height_3		Shoot Height_4	
**ANOVA**	Df	F	p	F	p	F	p	F	p	F	p
Block	4	0.9000	0.4800	1.7190	0.1790	0.6610	0.6250	0.3690	0.8285	0.6690	0.6200
Treat	6	12.9900	<0.0001	0.9900	0.4540	1.8800	0.1260	4.9090	0.0021	4.9840	0.0019
Residuals	24										
**Turkey's comparisons for Treatment factor, p-values**											
1T-1M			0.9029		1.0000		0.9995		0.9670		0.9992
2M-1M			0.9738		1.0000		0.9998		1.0000		0.9924
2T-1M			0.8860		0.9877		0.9991		0.9994		0.9987
3M-1M			0.0067		0.8944		1.0000		1.0000		0.9094
3T-1M			0.0043		1.0000		0.9985		0.8632		0.8815
C-1M			<0.0001		0.9433		0.2867		0.0032		0.0146
2M-1T			0.9999		1.0000		1.0000		0.9493		1.0000
2T-1T			1.0000		0.9990		1.0000		0.9989		1.0000
3M-1T			0.0907		0.7662		0.9981		0.9885		0.6868
3T-1T			0.0615		0.9999		0.9660		0.9998		0.6406
C-1T			<0.0001		0.8456		0.1399		0.0280		0.0049
2T-2M			0.9999		0.9976		1.0000		0.9983		1.0000
3M-2M			0.0496		0.8077		0.9989		1.0000		0.5467
3T-2M			0.0328		1.0000		0.9738		0.8234		0.5002
C-2M			<0.0001		0.8793		0.1532		0.0026		0.0028
3M-2T			0.0995		0.4816		0.9969		1.0000		0.6638
3T-2T			0.0677		0.9840		0.9566		0.9798		0.6172
C-2T			<0.0001		0.5780		0.1276		0.0092		0.0045
3T-3M			1.0000		0.9084		0.9997		0.9263		1.0000
C-3M			0.0759		1.0000		0.3451		0.0049		0.1641
C-3T			0.1109		0.9527		0.5684		0.0598		0.1882

Supplementary Table 3. Continued.

		N		Log_{10}(Ca)		√ Cd		Cu		K	
**ANOVA**	Df	F	p	F	p	F	p	F	p	F	p
Block	4	0.1250	0.9718	0.4430	0.7760	0.6150	0.6560	2.0100	0.1300	1.2880	0.3065
Treat	6	4.4360	0.0048	8.8360	<0.0001	9.7540	<0.0001	4.3850	0.0050	3.1840	0.0221
Residuals	21										
**Turkey's comparisons for Treatment factor, p-values**											
1T-1M			0.9007		0.9900		1.0000		0.0646		0.9999
2M-1M			0.2514		0.0203		0.0516		0.0365		0.9992
2T-1M			0.4073		0.4778		0.0082		0.6613		0.9997
3M-1M			0.0239		0.0007		0.0006		0.0568		0.7457
3T-1M			0.1507		0.9741		0.0045		0.9906		0.5834
C-1M			0.9742		0.0794		0.9998		0.0250		0.3842
2M-1T			0.8757		0.0041		0.0614		1.0000		1.0000
2T-1T			0.9681		0.1592		0.0099		0.7483		1.0000
3M-1T			0.2081		0.0002		0.0007		1.0000		0.5637
3T-1T			0.7276		0.6954		0.0055		0.2396		0.3943
C-1T			0.5196		0.0217		0.9999		0.9734		0.5455
2T-2M			0.9999		0.6042		0.9769		0.5847		1.0000
3M-2M			0.8230		0.6405		0.3373		1.0000		0.4879
3T-2M			0.9999		0.1206		0.9183		0.1489		0.3259
C-2M			0.0948		1.0000		0.2310		0.9949		0.6161
3M-2T			0.6528		0.0447		0.7949		0.6569		0.5260
3T-2T			0.9953		0.9284		1.0000		0.9632		0.3596
C-2T			0.1604		0.8246		0.0588		0.3573		0.5803
3T-3M			0.9315		0.0048		0.9038		0.2016		1.0000
C-3M			0.0101		0.6727		0.0054		0.9954		0.0427
C-3T			0.0566		0.3012		0.0365		0.0892		0.0220

Supplementary Table 3. Continued.

		Log_{10}(Mg)		Log_{10}(Mn)		Na		P		S		Log_{10}(Zn)	
**ANOVA**	Df	F	p	F	p	F	p	F	p	F	p	F	p
Block	4	2.3560	0.0868	1.0670	0.3980	0.7090	0.5950	0.5890	0.6740	0.4690	0.7575	0.8870	0.4887
Treat	6	12.5530	<0.0001	46.4500	<0.0001	1.2610	0.3170	5.5510	0.0014	4.3350	0.0054	7.2940	0.0003
Residuals	21												
**Turkey's comparisons for Treatment factor, p-values**													
1T-1M			0.9874		0.9274		0.4252		0.7969		0.1867		0.9970
2M-1M			0.2080		<0.0001		0.4112		0.0516		0.0079		0.1901
2T-1M			0.5398		<0.0001		0.9607		0.0112		0.2881		0.9997
3M-1M			0.0001		<0.0001		0.8387		0.0028		0.0050		0.0012
3T-1M			0.1960		<0.0001		0.9675		0.1007		0.6194		1.0000
C-1M			0.0004		0.6864		0.4258		0.9897		0.2732		0.6392
2M-1T			0.0499		<0.0001		1.0000		0.5395		0.7252		0.0641
2T-1T			0.1804		<0.0001		0.9245		0.1947		1.0000		0.9604
3M-1T			<0.0001		<0.0001		0.9960		0.0510		0.5116		0.0003
3T-1T			0.0464		<0.0001		0.9128		0.7396		0.9748		0.9908
C-1T			<0.0001		0.9935		1.0000		0.9987		1.0000		0.3510
2T-2M			0.9932		0.4608		0.9167		0.9908		0.5668		0.3439
3M-2M			0.0256		0.0086		0.9951		0.7369		0.9993		0.2045
3T-2M			1.0000		0.0910		0.9043		0.9999		0.2514		0.2373
C-2M			0.0484		0.0009		1.0000		0.3981		0.8826		0.9978
3M-2T			0.0063		0.3347		0.9994		0.9769		0.3712		0.0027
3T-2T			0.9911		0.9520		1.0000		0.9389		0.9962		1.0000
C-2T			0.0139		<0.0001		0.8808		0.1494		0.9998		0.8194
3T-3M			0.0275		0.8554		0.9991		0.5482		0.1487		0.0016
C-3M			1.0000		<0.0001		0.9836		0.0437		0.7162		0.1410
C-3T			0.0515		<0.0001		0.8678		0.5668		0.9749		0.7072

Supplementary Table 4. Results of the one-way ANOVA for the data from the rhizobox experiment. Df, degrees of freedom; F, F-statistic; p, p-value; DW, dry weight; $Log_{10}(X)$, Log_{10} transformation.

		Root Length_1		Root Length_2		Log_{10}(Root Length_3)		Log_{10}(Root Length_4)		Shoot Heigth_1	
**ANOVA**	Df	F	p	F	p	F	p	F	p	F	p
Treat	3	0.1300	0.9420	3.17	0.085	13.19	0.002	7.79	0.009	5.53	0.024
Error	8										
Total	11										
**Turkey's comparisons - intervals for the difference of means**											
		Lower	Higher	Lower	Higher	Lower	Higher	Lower	Higher	Lower	Higher
C - M		-145	115	-642	22	-1.15	-0.29	-1.30	-0.20	-15.26	0.26
C - P		-149	111	-482	182	-0.97	-0.10	-1.05	0.05	-10.09	5.42
C - T		-153	107	-548	116	-1.17	-0.31	-1.23	-0.13	-16.09	-0.58
M - P		-134	126	-172	492	-0.25	0.61	-0.30	0.80	-2.59	12.92
M - T		-138	122	-238	426	-0.45	0.41	-0.48	0.62	-8.59	6.92
P - T		-134	126	-398	266	-0.64	0.22	-0.73	0.37	-13.76	1.76

Supplementary Table 4. Continued.

		Shoot Heigth_2		Shoot Heigth_3		Shoot Heigth_4		Log_{10}(DW)		N		
**ANOVA**	Df	F	p	F	p	F	p	F	p	Df	F	p
Treat	3	11.73	0.003	12.63	0.002	19.6	<0.0001	23.01	<0.0001	2	0.63	0.562
Error	8									6		
Total	11									8		
**Turkey's comparisons - intervals for the difference of means**												
		Lower	Higher	Lower	Higher	Lower	Higher	Lower	Higher		Lower	Higher
C - M		-28.88	-5.78	-37	-6.0	-43	-10	-2.02	-0.80			
C - P		-20.55	2.55	-29	1.8	-36	-3.1	-1.62	-0.40			
C - T		-30.55	-7.45	-43	-12.7	-54	-22	-1.92	-0.70			
M - P		-3.22	19.88	-8	23.2	-9.3	23	-0.21	1.01		-0.35	0.34
M - T		-13.22	9.88	-22	8.7	-28	4.8	-0.51	0.71		-0.24	0.45
P - T		-21.55	1.55	-30	0.84	-35	-2.3	-0.91	0.31		-0.23	0.46

Supplementary Table 4. Continued.

		Ca		Cd		Log$_{10}$(Cu)		K		Log$_{10}$(Mg)	
**ANOVA**	Df	F	p	F	p	F	p	F	p	F	p
Treat	3	13.05	0.002	1.07	0.415	4.86	0.033	0.5	0.692	28.72	<0.0001
Error	8										
Total	11										
**Turkey's comparisons - intervals for the difference of means**											
		Lower	Higher	Lower	Higher	Lower	Higher	Lower	Higher	Lower	Higher
C - M		-0.211	0.706	-0.108	0.046	-0.038	0.300	-4.39	4.85	0.122	0.387
C - P		0.271	1.189	-0.116	0.038	-0.069	0.269	-4.39	4.85	0.145	0.410
C - T		0.276	1.193	-0.090	0.064	0.029	0.366	-3.04	6.20	0.233	0.498
M - P		0.024	0.941	-0.085	0.069	-0.200	0.138	-4.62	4.62	-0.110	0.156
M - T		0.029	0.946	-0.059	0.095	-0.103	0.235	-3.27	5.97	-0.022	0.244
P - T		-0.454	0.463	-0.051	0.103	-0.071	0.266	-3.27	5.97	-0.045	0.221

Supplementary Table 4. Continued.

			Log$_{10}$(Mn)			Na			P	
**ANOVA**	Df	p	F	p	Df	F	p	Df	F	p
Treat	3	<0.0001	15.01	0.001	3	1.06	0.425	3	31.06	<0.0001
Error	8				7			8		
Total	11				10			11		
**Turkey's comparisons - intervals for the difference of means**										
		Higher	Lower	Higher		Lower	Higher		Lower	Higher
C - M		0.387	-0.512	-0.138		-0.30	0.94		0.51	1.62
C - P		0.410	-0.325	0.049		-0.49	0.74		-0.36	0.75
C - T		0.498	-0.518	-0.144		-0.47	0.76		0.87	1.98
M - P		0.156	0.000	0.374		-0.74	0.36		-1.42	-0.32
M - T		0.244	-0.193	0.181		-0.72	0.38		-0.20	0.91
P - T		0.221	-0.380	-0.006		-0.53	0.57		0.67	1.78

Supplementary Table 4. Continued.

		S		Zn	
**ANOVA**	Df	F	p	F	p
Treat	3	1.79	0.227	4.48	0.04
Error	8				
Total	11				
**Turkey's comparisons - intervals for the difference of means**					
		Lower	Higher	Lower	Higher
C - M		-0.69	1.22	-22.4	20.4
C - P		-0.88	1.03	-9.2	33.5
C - T		-0.32	1.58	-1.7	41.1
M - P		-1.14	0.76	-8.2	34.6
M - T		-0.59	1.32	-0.7	42.1
P - T		-0.40	1.51	-13.8	28.9

Supplementary Table 5. Results of the comparison (one tailed paired t-test) between average percentage of new root length at right and left of the main root, in the rhizobox experiment, n=3.

Month	T	p
2nd month	1.98	0.093
3rd month	1.88	0.100
4th month	4.11	0.027
